# “Family doctors are also people”: a qualitative analysis of how family physicians managed competing personal and professional responsibilities during the COVID-19 pandemic

**DOI:** 10.1186/s12960-024-00901-4

**Published:** 2024-03-04

**Authors:** Sarah Spencer, Julia Lukewich, Emily Gard Marshall, Maria Mathews, Shabnam Asghari, Judith B. Brown, Thomas R. Freeman, Paul Gill, Samina Idrees, Rita K. McCracken, Sudit Ranade, Steve Slade, Amanda L. Terry, Jamie Wickett, Eric Wong, Richard Buote, Leslie Meredith, Lauren Moritz, Dana Ryan, Lindsay Hedden

**Affiliations:** 1 Faculty of Health Sciences, Simon Fraser University, 8888 University Drive, Burnaby, BC V5A 1S6 Canada; 2 Faculty of Nursing, Memorial University, 300 Prince Philip Drive, St. John’s, NL A1B 3V6 Canada; 3 Primary Care Research Unit, Department of Family Medicine, Dalhousie University, 1465 Brenton Street, Halifax, NS B3J 3T4 Canada; 4 Department of Family Medicine, Schulich School of Medicine & Dentistry, Western University, 1465 Richmond Street, London, ON N6G 2M1 Canada; 5 Family Medicine, Faculty of Medicine, Memorial University, 300 Prince Philip Drive, St. John’s, NL A1B 3V6 Canada; 6 Department of Family Practice, Faculty of Medicine, University of British Columbia, 5950 University Boulevard, Vancouver, BC V6T 1Z3 Canada; 7 The College of Family Physicians of Canada, 2630 Skymark Avenue, Mississauga, ON L4W 5A4 Canada

**Keywords:** Primary care, Family physicians, Pandemic, COVID-19, Personal responsibilities, Professional responsibilities

## Abstract

**Background:**

Family physicians (FPs) fill an essential role in public health emergencies yet have frequently been neglected in pandemic response plans. This exclusion harms FPs in their clinical roles and has unintended consequences in the management of concurrent personal responsibilities, many of which were amplified by the pandemic. The objective of our study was to explore the experiences of FPs during the first year of the COVID-19 pandemic to better understand how they managed their competing professional and personal priorities.

**Methods:**

We conducted semi-structured interviews with FPs from four Canadian regions between October 2020 and June 2021. Employing a maximum variation sampling approach, we recruited participants until we achieved saturation. Interviews explored FPs’ personal and professional roles and responsibilities during the pandemic, the facilitators and barriers that they encountered, and any gender-related experiences. Transcribed interviews were thematically analysed.

**Results:**

We interviewed 68 FPs during the pandemic and identified four overarching themes in participants’ discussion of their personal experiences: personal caregiving responsibilities, COVID-19 risk navigation to protect family members, personal health concerns, and available and desired personal supports for FPs to manage their competing responsibilities. While FPs expressed a variety of ways in which their personal experiences made their professional responsibilities more complicated, rarely did that affect the extent to which they participated in the pandemic response.

**Conclusions:**

For FPs to contribute fully to a pandemic response, they must be factored into pandemic plans. Failure to appreciate their unique role and circumstances often leaves FPs feeling unsupported in both their professional and personal lives. Comprehensive planning in anticipation of future pandemics must consider FPs’ varied responsibilities, health concerns, and necessary precautions. Having adequate personal and practice supports in place will facilitate the essential role of FPs in responding to a pandemic crisis while continuing to support their patients’ primary care needs.

**Supplementary Information:**

The online version contains supplementary material available at 10.1186/s12960-024-00901-4.

## Background

Family physicians (FPs) are an essential component of any pandemic response, with responsibilities ranging from surveillance and education to managing surge capacity, while continuing to provide routine primary care to their patients [1–9]. As the COVID-19 pandemic progressed in Canada, it became clear that the pandemic response was heavily reliant on FPs to support public health and acute care [6]. Though existing pandemic response plans in Canada articulated goals for primary care, these documents [3, 10, 11] tended to be clinically oriented and rarely recognised the complexity of FPs’ work context, which is often split across a variety of practice settings [12] and lacks formal connection to the broader health system.

Consequently, many community-based FPs did not receive sufficient or timely professional supports or resources [13], such as public health and COVID-19 information, practice guidance, and access to appropriate personal protective equipment (PPE) [14, 15]. This lack of support could leave FPs with pre-existing health conditions feeling vulnerable and those without concerned about how to contribute to the pandemic response while protecting their families from their work exposures [13]. FPs were also expected to make rapid adaptations in their practice, such as moving to virtual models of care, often with little support or prior experience [13, 16]. Frequently, the upheaval and lack of support FPs experienced professionally were reflected in broader social disruptions. In particular, just as FPs were being advised to minimise in-person visits [17–20] or asked to redeploy to support acute care or COVID-19 testing centres [6], schools and childcare centres across the country were being shut down [21–23].

Existing research has detailed the challenges that such closures and the pandemic overall have had on healthcare and essential workers more broadly [22–27], however there is little research that focuses specifically on FPs. The objective of our study was to explore the experiences of FPs during the first year of the COVID-19 pandemic to better understand how they managed their competing professional and personal priorities. By understanding how FPs’ personal experiences during the COVID-19 pandemic affected their professional activities (and vice versa), we aim to inform the provision of appropriate supports and planning for FPs during future pandemics.

## Methods

### Study design

We conducted a series of case studies in four regions of Canada (Vancouver Coastal health region in British Columbia, Eastern Health region in Newfoundland and Labrador, the province of Nova Scotia, and Ontario Health West) to document the anticipated and actual personal and professional responsibilities of FPs during the COVID-19 pandemic. Case studies included provincial policy scans to establish a chronology of FP roles and semi-structured interviews with FPs. Mathews et al. [28] have described the full study protocol, case study regions, and broader healthcare system context previously. This paper focuses on how FPs’ personal responsibilities influenced and were impacted by their professional practice and participation in the pandemic response.

### Sampling and recruitment

We used a maximum variation sampling approach to recruit FPs in each study region. We sought to include individuals representing a diverse group of characteristics, such as gender, urban and rural location, patient population, funding and practice models, and academic, hospital, or health authority affiliations. We used lists from family medicine and physician professional associations and faculty newsletters, provincial College of Physicians and Surgeons’ public listings, social media posts by study team members and collaborating organisations, and a snowball approach (where permitted) to recruit participants.

Regional Research Coordinators shared study information, an invitation to participate, and the consent form with prospective participants. Eligible FPs were clinically active or eligible to be clinically active in study regions. We excluded postgraduate medical residents, unlicensed international medical graduates, and FPs working in exclusively academic, research, or administrative roles.

### Data collection and analysis

Participants were scheduled for a 45 min to one hour telephone or Zoom videoconference (Zoom Video Communications) interview. The interviews were conducted between October 2020 and June 2021 and explored the varied expectations, roles, and responsibilities of FPs as the pandemic evolved, the facilitators and barriers that FPs faced in fulfilling these, and any personal or professional gendered experiences they encountered which affected their participation in the pandemic response. Our semi-structured interview guide (Additional file 1) was created by the regional principal investigators and pre-tested by the broader study team, which includes practising FPs and primary care and public health experts. Interviews were audio recorded, transcribed, and verified by the interviewer.

Transcripts were analysed using a thematic framework [29, 30]. At least two researchers independently reviewed and inductively coded a selection of transcripts from each region before meeting to develop a harmonised regional coding framework. Researchers then used their regional coding framework to code one transcript from each study region. Through a series of meetings, the researchers compared coding decisions and overlapping codes to develop a harmonised coding framework, resolving any conflicts through consensus. This harmonised framework was then used by researchers to code all transcripts in their study region using NVivo V.12 (QSR International).

### Ethics

We obtained ethics approval from the appropriate research ethics boards in each study region. All participants provided their written informed consent in advance of their interview, participation was voluntary, and responses have been anonymised. We use participant codes throughout our presentation of results which include a provincial abbreviation and an indication of the participant’s reported gender (man (M) or woman (W)).

## Results

We interviewed 68 FPs (Table 1), of whom 41 (60.3%) identified as women. Forty-six (67.7%) declared dependents, including children, elderly parents, or other family/community members for whom participants were responsible for providing care to varying degrees. Interviews were conducted between October 2020 and June 2021 and therefore covered FPs’ experiences during the early stages of the COVID-19 pandemic in Canada. Prominent themes emerged relating to FPs’ personal and professional responsibilities and concerns: (1) personal caregiving responsibilities; (2) COVID-19 risk navigation to protect family members; (3) personal health concerns; and (4) available and desired supports. The themes describe the issues FPs encountered in balancing their personal and professional responsibilities, followed by the solutions they relied upon versus those they would have liked to have had—for both COVID-19 and future pandemic scenarios; and in many cases, these reflect the natural progression of participants own self-reflections.

**Table 1:** Participant characteristics [*N*(%)]

	British Columbia	Newfoundland and Labrador	Nova Scotia	Ontario	Total
	*N* = 15	*N* = 12	*N* = 21	*N* = 20	*N* = 68
Sex/gender^a^					
Male/men	4 (26.7)	4 (33.3)	9 (42.9)	10 (50.0)	27 (39.7)
Female/women	11 (73.3)	8 (66.7)	12 (57.1)	10 (50.0)	41 (60.3)
Dependents					
Children Elderly parents Children and elderly parents	7 (46.7) 1 (6.7) 3 (20.0)	7 (58.3) 0 (0.0) 1 (8.3)	12 (57.1) 2 (9.5) 1 (4.8)	11 (55.0) 1 (5.0) 0 (0.0)	37 (54.4) 4 (5.9) 5 (7.4)
None reported	4 (26.7)	4 (33.3)	6 (28.6)	8 (40.0)	22 (32.4)
Community size^b^					
Rural	0 (0)	3 (25.0)	8 (38.1)	9 (45.0)	20 (29.4)
Small urban	0 (0)	0 (0.0)	0 (0.0)	1 (5.0)	1 (1.5)
Urban	15 (100.0)	8 (66.7)	13 (61.9)	8 (40.0)	44 (64.7)
Mix	0 (0)	1 (8.3)	0 (0.0)	2 (10.0)	3 (4.4)
Remuneration model					
Fee-for-service	6 (40.0)	5 (41.7)	7 (33.3)	4 (20.0)	22 (32.4)
Alternative payment plan^c^	9 (60.0)	7 (58.3)	14 (66.7)	16 (80.0)	46 (67.7)
Years in practice (mean)	16.9	16.3	15.4	18.7	16.9

^a^Gender was asked as an open-ended question and participants frequently responded using sex-based terminology, so we reflect that here by grouping ‘Sex/gender’

^b^Rural < 10,000 population, small urban = 10,000–99,999 population, urban > 1,000,000 population. Mix denotes where participants work in more than one community size

^c^Alternative payment plan includes all funding models outside of traditional and enhanced fee-for-service

### Personal caregiving responsibilities

Both men and women described the toll of their participation in the pandemic response on their personal caregiving responsibilities amidst the broader societal upheaval of the pandemic. For many participants, the pandemic did not shift the distribution of caregiving roles in their household. Rather, pandemic-induced changes reflected the existing distribution of responsibilities that each FPs’ family had established prior to the COVID-19 pandemic.

Where women carried the majority of household responsibilities pre-pandemic, they often felt the weight of the pandemic more so than others who more equally shared those roles with their partners: *“our roles as caregivers often falls to the woman—maybe that’s a sexist thing to say…. But it seems to me we get the brunt, the main responsibility for childcare—at least in my house”* [ON03W]. This was particularly the case where partners were either unwilling or unable to scale back on their professional duties, and could prompt women FP to limit their pandemic workforce participation: *“I felt like I had to cut back on my work just to keep things manageable at home with my personal life and with my kids and it felt like my partner really didn’t cut back on any of his roles”* [BC05W].

For many participants, every aspect of their lives became more challenging. As one participant noted, the increased difficulty—both at home and at work—was experienced equally between herself and her spouse: *“I feel really fortunate in that my spouse, we’re pretty equal in terms of how we share our workloads. So, my personal workload didn’t increase relative to his; both of our workloads just increased in every dimension”* [BC15W]. This heightened difficulty was particularly true during the initial stay-at-home closures when childcare centres and schools shut down. While this experience was not necessarily unique to FPs, the level of challenge participants faced was even greater in households comprising multiple essential workers: *“…childcare was a big challenge. Schools closed, I have three little kids, my husband’s also an essential worker. So childcare was a huge barrier for our family”* [NS02W]. Access to childcare, having older and more independent children, or a spouse who relieved childcare pressures could determine FPs’ ability to continue working at their pre-pandemic level or take on additional pandemic-specific roles. As one physician noted regarding their workload, *“When most of the rest of the world was slowing down, my world was ramping up”* [NL02W].

During school closures, FPs supervised and homeschooled their children using virtual platforms. For one household with two physicians, this required the FPs to split and schedule their clinic time around their children’s needs:

*…we have two kids, and for the first little bit with schools locked down it meant that we had to adjust all of our clinic schedules, so that if she was in clinic, I was at home and if I was at home, you know, and vice versa. And it really felt like we had to become teachers…* [ON14M]

For other FPs, homeschooling was an unrealistic expectation: *“The concept that you should somehow homeschool while you work, I thought that was crazy. The teacher literally emailed me and said, ‘You don’t seem to have logged in yet.’ I was like, ‘Nope, I’m not going to log in….’”* [NS15W].

With the rapid, widespread introduction of virtual modalities to support stay-at-home closures, some FPs saw patients from home. This was not always straight-forward, however, as their work required privacy for confidential patient consultations which could be challenging to achieve with other family members at home:

*So I would do my virtual care… I’d be at home for exactly that reason—to try to support, as I could, my kids and my family. […] so I tried to be in a physical isolation room as much as possible. And then come out between patients and say, ‘Hey, what can I do?’ and make you a quick sandwich, and then run back in. And then come back out again, and break up a fight, and go back in again.* [NS13M]

To continue their clinical practice and assist with the pandemic response, participants acknowledged the sacrifices that their partners made, including taking leaves from work in non-essential sectors, having only one essential worker in a family working or working full-time, and delaying the completion of academic studies.

### COVID-19 risk navigation to protect family members

While the pandemic introduced new complexities for all adults with personal caregiving responsibilities, FPs who provided in-person clinical care—sometimes with limited access to appropriate PPE—encountered an added stressor due to concerns surrounding the risk of exposure for themselves and their dependents. This was a frequent and explicit source of stress noted during the interviews and often revealed a conflict between competing identities of care provision—that of a physician versus that of a parent or child:

*So, it was just a real tangible stress and I think that I really internalised that because you never forget being a mom. […] I know that maternal feeling runs deep for me and I’m cuddling my kids in the middle of the night when they’re waking up and can’t fall back asleep, […] and so whatever I’ve been in touch with that day—the whole family’s getting it. And then you feel bad as a doctor for having faced that situation and now it’s like, am I a bad parent? Literally the conversations that doctor moms were having were, are you a bad parent for cuddling your kid?* [NL03W]

Yet, the measures FPs took to protect family members from any COVID-19 exposures could be another source of stress. One FP who lived in the basement, separate from his family to limit exposure, noted that some of these measures were not sustainable over a prolonged period:

*I didn’t want to be the guy that brought COVID to my family […] I was isolated from my family. I was in full work mode and when I’d come home late at night, they’d come down and visit with me from the opposite side of the barrier and that was not good for my mental health, not good for my family’s health. So, we eventually abandoned that and sort of said, there has to be some sort of compromise here, and I went more to the strip and shower approach that my colleagues were doing.* [ON04M]

These decisions were not without impact for FPs or their family members. Amidst PPE shortages and increased workload, participants knew that their families were worried about both their physical and emotional wellbeing. FPs similarly faced the emotional toll of having to minimise contact with family:

*So my sister lives two hours away. She’s also a family doctor who was working at the ER last year […] her husband stays home full-time with their three children. So my ten year old went and lived in their house […] from March break until the end of May […] And two households with two doctors in them seemed like a bad idea to mix us up […] So I stayed home, worked full-time and I didn’t see my youngest daughter*. [NS15W]

### Personal health concerns

FPs who identified as young and healthy acknowledged the importance of continuing to work and see patients in-person, while also expressing fears about their health and level of protection against COVID-19: *“I told myself that this is my duty to be there […] and I kept my fears just at the back corner”* [NS12W]. This duty of care expressed by FPs often superseded their personal concerns:

*[I knew] that I was going to be at risk and that I couldn’t change that. I couldn’t abandon my patients and I also couldn’t tell everybody to go to emerg with all their concerns […]. I think early March I knew that it was going to be a long-haul and I knew that I wasn’t going to be protected. And I would still have to deliver care.* [NL11W]

These concerns were heightened for FPs with chronic illnesses, who were immunocompromised, or older. As one participant noted, *“I’m the old guy without young children and able to probably do some of that backfilling. But on the age side, I’m also at greater risk, being 65—how do we take [that] into consideration?”* [NS01M]. This was echoed by another participant after inquiring about supports for FPs:

*And at the time, the concern was, ‘well what about the older doctors with comorbidities and lack of protection? Is there any exemption or support for us?’ And [the Medical Officer of Health’s] answer, I remember, was just, ‘No.’ […] It was just unbelievable, I thought, we’re just cogs in a wheel. They’re talking about vulnerable patients, older patients with comorbidities. Well, many of our doctors are also older, like in their 70s or 60s, and with comorbidities. So, what about us?* [BC10W]

### Available and desired supports

Personal supports available to FPs varied by study region, reflecting provincial-level pandemic responses. In British Columbia, FPs expressed gratitude for the decision to prioritise keeping schools open:

*I have been deeply appreciative of the approach BC’s taken, whatever criticism other people may have about trying to keep schools opened at all costs. I know that’s been highly controversial … It [school closure] was a disaster for our family last year, it was not good at all and I’m sure we’re not alone.* [BC13W]

In Nova Scotia, participants were disappointed by the lack of consideration for FPs and other essential workers’ childcare needs, with one FP expressing particular frustration at the Premier’s suggestion during a daily briefing [31] that these needs were being taken care of “organically” by nearby family members, friends, and neighbours, as this assumes that all FPs had these local supports available to allow them to continue working and contributing to the pandemic response:

*They talked a lot about families supporting each other and helping young families take care of kids. And I’m listening to this and thinking, I don’t know who you’re talking about. You’re talking about families that are from Nova Scotia, who have generations of family in Nova Scotia […] So that’s none of the immigrants, that’s none of the single parents, that’s none of the people who have moved here. […] And it was a very difficult message to hear from two older white men [the Premier and the Chief Medical Officer of Health].* [NS15W]

Other participants from Nova Scotia echoed this sentiment, pointing to the need to provide a more equity-oriented and *“gendered response”* to the pandemic that supported FPs professionally and “*in the context of their family and the stress they’re going through, and we didn’t do that at all”* [NS13M].

In Ontario, participants provided little indication of available supports beyond some medical students offering childcare. In Newfoundland and Labrador, participants suggested that care for children was available but limited to FPs providing in-person care: “*So, if you were working outside the home, there was supports. But, as far as I know, if you were working in your home, there’s not”* [NL10W].

More often, participants noted their reliance on informal supports to manage their personal responsibilities and continue working throughout the pandemic. Family and community supports were the most prominently mentioned, with many participants—both men and women—acknowledging their spouse: *“I think having a spouse who looked after the family was the number one thing that allowed me to do what I did.”* [ON13M]. There were even instances where spousal support included supporting FPs professionally. Multiple participants noted that their spouses stepped in to provide them clerical support which, in one case, involved training on their electronic medical record and signing a confidentiality agreement with the health authority [NL12W].

Participants also cited the importance of a strong practice community to provide professional support. This was especially crucial for FPs who were limited to virtual practice due to their own clinical vulnerabilities: *“I’m strictly virtual care and the docs I’m filling in for know that and provide back-up for me so that my patients still get seen if they need to be”* [NL10W].

When asked what FPs would need to participate fully in a pandemic response, the variety of supports articulated reflected the varied experiences and personal circumstances of FPs. Certainly, childcare was a prominent request, particularly childcare that reflected the idiosyncrasies of FPs’ schedules: *“So, having affordable, reliable childcare and flexible childcare. And so that’s the other piece that is more challenging perhaps for physicians is that often the traditional daycare, 8–5 or whatever, isn’t always helpful”* [BC07W].

For other FPs, there was a desire for schedule flexibility, noting that this requires administrative and colleague support, and a funding model that provides some *“leeway in your deliverables to be able to do that”* [NS11W]. Though some FPs felt fortunate that they had financial privileges that other families may not, enabling them to retain or hire caregivers or cut back on their work during the pandemic, others felt financially compelled to continue working to support their families. This was certainly a concern for one fee-for-service FP who struggled to support her mother in caring for her father with dementia: “*it’s difficult for me […] I have to consider the financial ramifications to my more immediate family if I was to take time off work—which would be unpaid—in order to provide homecare or respite for mom”* [NL07W].

## Discussion

Familial roles and personal circumstances including personal caregiving responsibilities, COVID-19 risk navigation to protect family members, and personal health concerns complicated FPs’ ability to deliver routine primary care and participate in the pandemic response. Many of the experiences articulated by FPs during our interviews echo those of other healthcare professionals and essential workers, especially with respect to the added stress resulting from school and childcare centre closures [21–23], minimising the risk of exposing family to COVID-19 through their work [26, 32–34], and a lack of equity-informed formal supports to help manage conflicting personal and professional responsibilities [24, 27, 35].

FPs, however, did articulate a distinct experience with the rapid introduction of virtual care to facilitate stay-at-home closures early in the pandemic and minimise COVID-19 exposure risks throughout [16]. For FPs who employed this new modality to work from home, finding private spaces to hold confidential conversations with patients could be a challenge. This was particularly the case for FPs with younger children, who had to support homeschooling, or who did not have a partner who took on additional household responsibilities. These experiences informed the ardent call from participants for childcare supports. They reflected the difficulty of prolonged school and childcare centre closures, as well as the loss of informal sources of childcare due to both the need to protect high-risk family members who would ordinarily have provided care and the formation of social bubbles [21, 25, 36]. They also reflected regional variation in formal supports available to FPs amidst stay-at-home closures [37, 38], and existing research that documents FPs’ perceived exclusion from formal pandemic supports relative to frontline or hospital-based staff [24].

For FPs with heightened personal health concerns, including those who were immunocompromised and/or older, virtual modalities did provide them opportunities to continue working and seeing patients throughout the pandemic while minimising their exposure risks. There are limitations, however, to the utility of virtual modalities in providing care to patients and not all patients are comfortable with or have access to the requisite technologies [39]. Recognising this limitation, many FPs continued to provide in-person care throughout the pandemic but did so in the face of COVID-19 exposure concerns for both them and their family members [13]. These concerns were most prominent early in the pandemic when less was known about COVID-19 transmission and as FPs struggled to secure appropriate PPE, particularly those without access to government PPE supplies [40–42]. Though the FPs we interviewed displayed resilience and resourcefulness in navigating these health concerns and PPE deficiencies, quicker and easier access to PPE for all FPs in future pandemics should help ameliorate their personal and family health concerns and better support their participation in a pandemic response [40].

While many of the women with whom we spoke did not feel that their domestic responsibilities had increased relative to their partners’, this tended to reflect the distribution of labour in each family unit that had been established prior to the pandemic. For women who were already providing more child or parental care, or those without partners and local supports to attenuate those demands, the pandemic certainly amplified their unpaid care roles [22, 25, 32, 33]. Meanwhile, men who described the pressures of domestic responsibilities during the pandemic reflect the impact of broader societal disruptions on all personal caregivers, as well as reports of increases in men’s participation in childcare during stay-at-home closures [43].

The impact of personal responsibilities and circumstances on FPs’ ability to participate in a pandemic response occur amid wider concerns about primary care funding and access [44–47], including FP access to disability insurance and locum coverage [48]. Collectively, these individual and system level concerns are believed to contribute to the burnout, turnover, and disillusionment with family medicine in general [49–51]. Additional analyses suggest that inter-professional team-based and larger group practices may provide collegial support to FPs while alternative payment models (rather than fee-for-service) can alleviate financial concerns, allowing FPs to take time off [51, 52]. However, the diversity of FP experiences and desired supports we heard during interviews may reflect the challenge in establishing supports to meet everyone’s needs. Regardless, our findings highlight the need to address more comprehensive employment supports in future primary care reforms to strengthen the availability of primary care throughout a pandemic, and more broadly.

### Limitations

We interviewed FPs in four regions of Canada between October 2020 and June 2021; accordingly, our findings may not reflect the experiences of FPs in other jurisdictions or during later stages of the pandemic. We were unable to recruit any FPs who left practice during the pandemic due to personal circumstances and thus those perspectives are absent from our analysis. While we specifically asked all participants about the impact of gender and personal responsibilities on their participation in the pandemic response, personal health concerns and FPs’ pandemic-related risk mitigation efforts were not included in our interview guide and emerged organically during some interviews. Further, while interviewers ask for participants’ gender, responses frequently employed sex-based terminology (i.e. male or female). Though we recognise the distinction and do not want to conflate participants’ gender identity with their biological sex, we have grouped ‘sex/gender’ in our participant characteristics and included an indication of gender in each participant code. Finally, interviews are susceptible to social desirability and recall bias [53, 54].

## Conclusion

This analysis adds the voices of FPs to existing literature on the experiences of essential and healthcare workers during the COVID-19 pandemic in Canada, identifying both overlapping and distinct experiences. Notably, for FPs to contribute fully to a pandemic response, future pandemic plans must recognise the complexity of FPs’ experiences—in both their professional and personal lives. Where stay-at-home closures and homeschooling are required, childcare supports must be made available to all essential workers. Governments and health systems need also to make PPE readily available to healthcare workers in all settings to protect physicians themselves, and to help physicians protect their family members as they contribute to a pandemic response. Conversely, failure to appreciate their pandemic roles, the contexts in which they practise, as well as their competing personal and professional responsibilities leaves FPs and their practices unsupported. Comprehensive planning in anticipation of future pandemics must consider FPs’ varied responsibilities, health concerns, and necessary protective measures. Having adequate personal and practice supports in place will facilitate the essential role of FPs in responding to a pandemic crisis while continuing to support their patients’ primary care needs.

### Supplementary Information

**Additional file 1: **Interview guide.

## Data Availability

The datasets generated and analysed during the current study are not openly available to maintain the anonymity and confidentiality of participants but may be available from the corresponding author on reasonable request.

## Abbreviations

FP: Family physician
PPE: Personal protective equipment
