# Supplementary material for: “Family doctors are also people”: a qualitative analysis of how family physicians managed competing personal and professional responsibilities during the COVID-19 pandemic
Source: Hum Resour Health. 2024 Mar 4;22:18. doi: 10.1186/s12960-024-00901-4 (PMC10913223; doi:10.1186/s12960-024-00901-4)
Supplement: Supplementary file 1 — Additional file 1: Interview guide. [file 12960_2024_901_MOESM1_ESM.docx]

Additional File 1. Interview Guide

In this study, we want to gain a better understanding of the roles of family physicians during a pandemic. By roles, we mean specific tasks and/or responsibilities that family physicians are asked or required to do during the stages of the pandemic.

First, I would like to ask some general background questions.

1. How long have you been practicing as a family physician?
2. In which communities do you currently practice and how would you describe them in terms of urban or rural?
3. What is your practice model? How are you paid?
4. Can you tell me about the nature of your current practice in terms of where you work, for example, do you provide care in a community-based practice? ED? Long term care home? Hospital as a hospitalist? Home visits?
5. Do you belong to organized networks or physician groups? Which ones?
6. Do you have privileges at any hospital or other facility?
7. Do you have any contractual or other obligations to any health care organizations (e.g. local hospital, medical school, long term care home or other facility)?
8. What is your gender?
9. Do you routinely care for dependent family members?

In the next set of questions, I’d like to focus on the period from January to mid-March 2020. During this time, we first started to hear about COVID19 and cases were starting to show up in Canada.

1. During the PRE-CLOSURE period, could you describe what ACTUAL roles or functions you carried out for patients who had (or were suspected to have) COVID19 as well as your other patients?
   - *[Probe based on responses to first set of questions]*: community based practice, LTC, ED, hospital, other
2. Can you tell me about what supports were available to you to help carry out these roles? What barriers did you experience?
   - Probe: access to PPE, funding, communications with networks/groups/hospital,
3. Looking at the list of PROPOSED roles during the pre-closure stage, what supports would be needed to enable you to carry out these roles? What barriers did you experience?
   - Probe: access to PPE, funding, communications with networks/groups/hospital
4. Are there other roles that family physicians could or should have played during the PRE-CLOSURE stage? What supports would be needed to carry out those roles?
   - Probe: roles at different facilities
   - Probe: access to PPE, funding, communications with networks/groups/hospital
5. Is there anything else about this stage that you would like to share with us?

Now, let’s consider the CLOSURE Stage, from mid-March to mid-May. During this time, schools and most businesses were closed and physicians were advised to close practices except for essential visits.

1. During the CLOSURE period, could you describe what ACTUAL roles or functions you carried out for patients who had (or were suspected to have) COVID19 as well as your other patients?
   - Probe: access to PPE, funding, communications with networks/groups/hospital
2. Can you tell me about what supports were available to you to help carry out these roles? What barriers did you experience?
   - Probe: access to PPE, funding, communications with networks/groups/hospital
3. Looking at the list of PROPOSED roles during the CLOSURE stage, what supports would be needed to enable you to carry out these roles? What barriers did you experience?
   - Probe: access to PPE, funding, communications with networks/groups/hospital
4. Are there other roles that family physicians could or should have played during the CLOSURE stage? What supports would be needed to carry out those roles?
   - Probe: roles at different facilities
   - Probe: access to PPE, funding, communications with networks/groups/hospital
5. Is there anything else about this stage that you would like to share with us?

Now, I’d like to consider the PHASED RE-OPENING Stage, from mid-May to today. During this time, many businesses re-opened in some form. School opening plans were introduced and physicians were advised to limit in-person care to essential visits.

1. During the PHASED RE-OPENING period, could you describe what ACTUAL roles or functions you carried out for patients who had (or were suspected to have) COVID19, as well as your other patients? What barriers did you experience?
   - Probe: access to PPE, funding, communications with networks/groups/hospital
2. Looking at the list of PROPOSED roles during the PHASED RE-OPENING stage, what supports would be needed to enable you to carry out these roles? What barriers did you experience?
   - Probe: access to PPE, funding, communications with networks/groups/hospital
3. What other roles do you feel family physicians could or should have played during the PHASED RE-OPENING stage? What supports would be needed to carry out those roles?
   - Probe: roles at different facilities
   - Probe: access to PPE, funding, communications with networks/groups/hospital
4. Is there anything else about this stage that you would like to share with us?
5. To date we have been able to avoid a scenario where the emergency departments and hospitals are overwhelmed by COVID19 cases. If this were to happen, what additional roles should family physicians have?
   - Probe: roles at different facilities
6. What supports would be needed to allow family physicians to fulfil these roles? What barriers exist to these roles?
   - Probe: Access to PPE, funding, communications with networks/groups/hospital

For the final set of questions, I’d like to switch gears a bit.

1. Can you tell me about other non-physician responsibilities you have in your life?
2. How do your non-physician responsibilities influence the roles that you are able to play in a pandemic? What supports are needed to allow family physicians with other responsibilities to fulfill pandemic roles? What barriers did you experience?
3. Thinking of your gender… Does your gender influence the roles that you are able to play in a pandemic? What supports are needed to allow all genders to fulfill these roles? What barriers did you experience?

1. Should we go through additional pandemic stages and family physician roles evolve, may we contact you about doing another interview in the future about those additional stages and roles? You can decide whether you want to participate at that time.

Those are all the questions I have. Is there anything you would like to add?
